# Erratum

**DOI:** 10.1590/1518-8345.0000.3645

**Published:** 2022-05-25

Regarding the article “Psychometric properties of the adapted instrument European Health Literacy Survey Questionnaire short-short form”, with DOI number: 10.1590/1518-8345.4362.3436, published in the Rev. Latino-Am. Enfermagem. 2021;29:e3436, page 3:

Where was written:

“According to the authors of the instrument, the final score values classify individuals according to three levels of HL: inadequate (≤2); problematic (>2 and ≤3); and sufficient (>3)^(7,9-10)^.”

Now read:

“According to the authors of the instrument, the final score values classify individuals according to three levels of HL: inadequate (≤2); problematic (>2 and <3); and sufficient (≥3)^(7,9)^.”

Regarding the article “Use of the prone position in pregnant women with COVID-19 or other health conditions”, with DOI number: 10.1590/1518-8345.5181.3494, published in the Rev. Latino-Am. Enfermagem. 2021;29:e3494, page 1:

Where was written:

“Original Article”

Now read:

“Review Article”

Regarding the article “Psychopathological symptoms and work status of Southeastern Brazilian nursing in the context of COVID-19”, with DOI number: 10.1590/1518-8345.5768.3518, published in the Rev. Latino-Am. Enfermagem. 2022;30:e3518, page 1:

Where was written:

“Darcio Tadeu Mendes^7^ https://orcid.org/0000-0001-5180-1594”

Now read:

“Darcio Tadeu Mendes^7^ https://orcid.org/0000-0001-6059-9308”

